# Antimycobacterial and Antifungal Activities of Leaf Extracts From *Trichilia emetica*

**DOI:** 10.1155/sci5/8784390

**Published:** 2024-12-09

**Authors:** Lydia Chenesai Mugayi, Stanley Mukanganyama

**Affiliations:** ^1^Department of Biotechnology and Biochemistry, University of Zimbabwe, Mount Pleasant, Harare, Zimbabwe; ^2^Department of Therapeutics, Natural Products Unit, Wilkins Hospital Block C, Cnr J. Tongogara and R. Tangwena, The African Institute of Biomedical Research and Technology (AiBST), Harare, Zimbabwe

**Keywords:** antifungal activity, antimycobacterial activity, mode of action, *Trichilia emetica*

## Abstract

The global problem of infectious and deadly diseases caused by microbes such as candida and mycobacteria presents major scientific and medical challenges. Antimicrobial drug resistance is a rapidly growing problem with potentially devastating consequences. Various pathogens can cause skin infections, such as bacteria, fungi, and parasites. Antimicrobial resistance has caused the urgency to seek alternative treatment options from available natural resources. Plant-derived medicinal compounds can provide novel alternative treatment avenues against pathogenic microbes. The objective of this study was to determine the antimycobacterial and antifungal activity of leaf extracts of *Trichilia emetica* against *Mycobacteria smegmatis*, *Mycobacteria aurum*, *Candida tropicalis,* and *Candida albicans*. The leaf extracts were prepared using hexane, ethyl acetate, acetone, dichloromethane (DCM), methanol, ethanol, water, DCM:methanol, and 70% ethanolic aqueous solution. The microbroth dilution was used to determine the minimum inhibitory concentration (MIC) of each extract against the four test organisms. The mode of action by which these extracts inhibit growth was also investigated. The effects of the extract on the cell wall of *C. tropicalis* were determined using the sorbitol assay. The effects of the extracts on the membrane integrity of the test organisms were determined using propidium iodide, which binds to nucleic acids, and the Bradford reagent, which reacts with proteins. The ethyl acetate and 70% ethanolic aqueous extracts were most potent against the organisms tested with MICs ranging from 125 to 1000 μg/mL. However, the two extracts did not inhibit the growth of *C. tropicalis* in the presence of sorbitol. The extracts caused the leakage of nucleic acids and proteins in *C. tropicalis* and *M. smegmatis* only and not in *M. aurum.* It is concluded that the leaf extracts of *T. emetica* have antimycobacterial or antifungal activities. The disruption of cell membranes resulting in protein and nucleic acid leakage could be the plant's possible mode of action.

## 1. Introduction

Skin and soft tissue infections involve microbial invasion, and it has different presentations and severity. Most skin and soft tissue infections are caused by bacterial, viral, and fungal pathogens [1]. In humans, mycobacterial diseases are usually due to *Mycobacterium tuberculosis* and *Mycobacterium leprae*. However, disseminated infections caused by *Mycobacterium smegmatis* and *Mycobacteria aurum* are commonly related to immunosuppression [2, 3]. The cases of infections caused by *Candida* species have increased considerably over the past years. These cases are mainly due to the rise of the AIDS/HIV epidemic, an increasingly aged population, a higher number of immunosuppressed people, and the increased use of in-dwelling medical devices [4]. *Candida albicans* is the main cause of candidiasis but, non-*Candida albicans* species such as *Candida tropicalis*, *Candida glabrata*, and *Candida parapsilosis* have now been identified as human pathogens. Resistance of microorganism to drugs has become an increasingly recognized phenomenon [4].

Natural products in the form of standardized extracts or pure compounds provide many opportunities for novel drug leads due to the availability of chemical diversity [5, 6]. Plants represent an almost inexhaustible source of chemical compounds that have great potential use in medicines and other applications. Many bioactive compounds, such as alkaloids, flavonoids, phenols, terpenoids, tannins, steroids, glycosides, and volatile oils, are found in plants [6]. Natural products and their derivatives represent more than 50% of the drugs used. About 85% of traditional medicines use plant extracts, and the WHO has estimated that about 3.5–4 billion people worldwide rely on traditional medicines as a source of their primary health care [7]. Herbal medicines are still widely used, as they have been the basis of health care throughout the world since the earliest day of humankind [8]. Humans have acquired the skill of herbal healing through selecting plants deliberately or by accidental discovery [9].

Herbal medicines gradually lost popularity, as rapid synthetic drugs that have therapeutic action took over [10]. However, there has been a universal shift from synthetic to herbal medicines that can be said to be the “Return of nature” [11]. The increase in diseases and ailments has brought an urgent need for the development of safer drugs for the treatment of diseases. Consequently, plants can be used as the main source of medicine, not only as isolated phytochemicals to be distributed as standardized doses but also as crude drugs for the people [12]. Studies by Kebebew [13] showed that there is an urgent need to study and record the vast knowledge of medicinal plants that exists within the people before it is lost with subsequent generations. Therefore, it is of great importance for the study of plants to support traditional medical knowledge [14]. However, the pharmacological activity and chemical composition of most plants that have been used in traditional medicines have not been investigated [15].

*T. emetica* is a plant native to the open woodland of Africa. It is commonly known in the local vernacular language *Shona* as *Muchikiri,* belongs to the family Meliaceae. The name of the species refers to the three-lobed fruits, and the name of the genus refers to the emetic properties it carries. Some of the traditional uses of this plant in southern Africa are treatment of convulsion fever, jaundice, epilepsies, scabies, and respiratory and skin infections [16]. *T. emetica* seeds can be crushed and mixed with vegetables to add flavor [17]. Root bark has purgative and emetic effects, has been used against fever and leprosy, and promotes fertility in women [17]. *T. emetica* seeds can be crushed and mixed with vegetables to add flavor [18]. Leaves are used against scabies, malaria, hypertension, burns, and cutaneous and mouth infections. In Eastern Africa, the fruit has been used against cirrhosis, internal worms, hepatitis, poisoning, asthma, and dysmenorrhea [19]. The stem bark is used against cough, bronchial trouble, and some venereal diseases [16]. There are claims that the leaves have soporific effects, which means that they cause sleep when placed in bed at night. Hot infusions of the leaves of *T. emetic*a have been used to heal and soothe bruises [20, 21]. Some studies have shown the presence of triterpenes, resins, and tannins in the *T. emetica* bark [22–24]. In this study, *T. emetica* was selected as it has been used in Africa to treat different diseases, including respiratory and skin infections [16]. The microorganisms used in this study are known to cause skin and respiratory infections [2–4]. The aim of this study was to investigate the antimycobacterial (*M. aurum and M. smegmatis*) and antifungal (*C*. *albicans* and *C. tropicalis*) activity of the leaf extracts of *T. emetica*. Although there have been reports on the anticandidal activity of *T. emetica*, there is no literature on the anticandidal activity against *C. tropicalis* and mycobacteria [16]. The study furthermore explored the possible biochemical mode of action of the most potent extracts.

## 2. Materials and Methods

### 2.1. Chemical and Reagents

The chemicals used were purchased from Sigma-Aldrich (Darmstadt, Germany). The solvents used were dimethyl sulfoxide, hexane, ethyl acetate, acetone, ethanol, methanol, and dichloromethane (DCM). Other chemicals used included Sabouraud dextrose agar (SDA), tryptic soy agar, (2-(4-iodophenyl)-3-(4-nitrophenyl)-5-phenyl-2H-tetrazolium chloride) INT, Middlebrook 7H9 base, Middlebrook agar, Coomassie brilliant blue G-250, propidium iodide (PI), sorbitol, and sodium dodecyl sulfate.

### 2.2. Plant Collection and Authentication

The leaves of *T. emetica* were collected at the University of Zimbabwe (17°47′03.0″S + 31°02′55.4″E), and the authentication was done by Mr. Chapano of The National Botanical Garden (Harare, Zimbabwe). A voucher reference specimen was kept at the Biomolecular Interaction Analyses Laboratory in the Department of Biotechnology and Biochemistry at the University of Zimbabwe.

### 2.3. Preparation of Plant Extracts

The leaves were dried in a hot-air incubator (Labotec Co., Cape Town, South Africa) at 40°C for 1 week. Traditional mortar and pestle were used to grind the leaves into fine powder. The solvents of different polarities used were hexane, DCM, ethyl acetate, acetone, ethanol, methanol, water, 70% ethanolic aqueous, and 50:50 DCM:methanol. After using each solvent to individually extract from 100 g of powder, the filtrate was discarded following each extraction. A volume of 200 mL of each solvent was used for microextraction. Microextraction is defined as an extraction technique in which the volume of the extraction phase is very small compared to the volume of the sample and the extraction of analytes is not exhaustive [25]. Whatman1 filter paper (Sigma, Taufkirchen, Germany) was used for filtration to obtain the extract. The extraction was repeated until the filtrate became clear. The water filtrate was dried in a rotor vapor and then left to completely dry in a hot-air blowing oven, while the other filtrates were left under a fan to allow evaporation of the organic solvents. The dried extracts were placed in tightly sealed glass vials and stored at room temperature.

### 2.4. Gas Chromatography–Mass Spectrometry (GC–MS) Analysis

The GC-MS analysis was performed using a capillary column called HP-5 MS, which had dimensions of 25-m length and 250-*μ*m diameter, with a thickness of 0.25 *μ*m [20]. The instrument setup consisted of an Agilent 6890 TM GC coupled to a Waters GCTTM Premier mass spectrometer. Helium gas was used as the carrier gas, flowing at a constant rate of 1 mL/min. The oven temperature was initially set to 50°C and maintained for 6 min. Then, it was increased at a rate of 4°C per minute until it reached 300°C, where it was held for 30 min. The leaf samples (ethyl acetate and 70% ethanolic aqueous extracts) dissolved in chloroform and ethyl acetate, respectively, were manually injected at a temperature of 250°C. The mass spectra were obtained using electron ionization (EI) with an electron energy of 70 eV. The chromatogram was generated using the OpenChrom program. Compound identification from the generated chromatogram was performed by comparing the mass spectra with the NIST 17 MS database and the MS search program Version 2.3.

### 2.5. Growth of Microbial Cells

*M. smegmatis* mc^2^ 155 and *M. aurum* A+ were obtained from Professor D. Steenkamp of the Department of Clinical Laboratory studies and Professor Peter Smith of the Department of Pharmacology, respectively (University of Cape Town) [26]. Mycobacteria were kept as glycerol stocks at −80°C. The microbial stocks were first cultured in Middlebrook broth supplemented with casein for 24 h followed by plating on Middlebrook agar. Cells were grown for 20 h in Middlebrook broth at 37°C, 100 rpm (Lab Companion Incubator SI 300 Jeio Tech Co. Ltd., Seoul, Korea) and standardized using 0.5 M McFarland at 2 × 10^6^ CFU/mL for antimicrobial susceptibility tests. *Candida albicans* (NCPF 3255) was obtained from Sigma-Aldrich (Steinheim, Germany). The clinical strain of *Candida tropicalis* was obtained from the Department of Medical Microbiology of Parirenyatwa Hospital in Harare, Zimbabwe. These were inoculated on Sabouraud dextrose broth and incubated for 20 h at 100 rpm in the Lab Companion Incubator SI 300 (Jeio Tech Co. Ltd., Seoul, Korea). The mycobacterial/fungal suspensions were prepared in sterile media to obtain a turbidity of 0.5 Mac Farland scale, equivalent to 1.5 × 10^8^ CFU/mL at OD 600 nm. These cells were then standardized to 2 × 10^6^ CFU/mL [27, 28].

### 2.6. Determination of the Minimum Inhibition Concentrations (MICs) and Minimum Bactericidal Concentrations (MBCs)

The microbroth dilution method was carried out following the European Committee of Antimicrobial Susceptibility Testing (EUCAST) protocol with alterations to determine the antimicrobial activity of the extracts [29]. Two microbroth dilution methods were used to determine the MIC, which were (i) using only tetrazolium salts and (ii) measuring turbidity by a microplate reader. Preliminary screening of the antimicrobial activity of the plant extracts was first done using 2-(4-iodophenyl)-3-(4-nitrophenyl)-5-phenyl-2H-tetrazolium chloride which is a tetrazolium salt. Tetrazolium salts act as an electron receptor in the electron transport chain of microbes, and during the reduction process, they change from being a colorless compound to a colored insoluble formazan. Dilutions were carried out to obtain a stock solution of 4000 μg/mL of extract, 25 μg/mL miconazole, and 50 μg/mL rifampicin. Serial dilutions of extracts and antibiotics were carried out directly on the microtiter plate to obtain a final concentration of 1000 μg/mL extract, 12.5 μg/mL miconazole, and 25 μg/mL rifampicin. The extracts were tested at four concentrations: 1000 μg/mL, 500 μg/mL, 250 μg/mL, and 125 μg/mL. In each of the wells, 100 μL of the standardized microbial cells (2 × 10^6^ CFU/mL) was added to achieve a final concentration of 1 × 10^6^ CFU/mL [28, 30]. Each experiment was done once with 4 replicates. Plates were incubated at 37°C overnight (Lab Companion Incubator SI 300, Jeio Tech Co Ltd, Seoul, Korea). The viable cells after exposure to the extracts were further evaluated by adding 20 *μ*L of 2-(4-iodophenyl)-3-(4-nitrophenyl)-5-phenyl-2H-tetrazolium chloride to each of the wells of the plate followed by incubation at 37°C for 1 h. The wells containing viable cells turned the colorless INT to a red color [31]. The growth of microbial cells in each well was determined by observing and comparing the test wells with the positive and negative controls. Antimicrobial activities were observed as wells without any microbial growth. The MIC was the lowest concentration of the extracts that prevented the visible growth of microorganisms. The ethyl acetate and 70% ethanolic aqueous extracts were further subjected for the micro-broth dilution method again, and the activity was then measured by a microtiter plate reader. Cell densities were measured at 590 nm using a microplate reader (Tecan Genios-Pro microplate reader, Grödig, Austria) before incubation and after incubation to determine the bioactivity of the extracts. MBCs and minimum fungicidal concentrations (MFCs) were determined by dipping the inoculation loop in each well without visible growth and using it to streak on Middlebrook agar for mycobacteria and SDA for fungi. MBCs or MFCs were determined as the lowest concentration that did not have any microbial growth on the agar after overnight incubation at 37°C [32].

### 2.7. Determination of the Effects of Potent Extracts on Fungal Cell Wall

The ethyl acetate and the 70% ethanolic aqueous showed potent antifungal activity and, therefore, these extracts were investigated to determine their effects on the cell wall of *C. tropicalis.* The MIC of the extracts was determined in *C. tropicalis* using the micro-broth dilution method in 96-well plates. Sorbitol was added to the Sabouraud dextrose broth as an osmotic protector (final concentration 0.8 M). The plates were incubated at 37°C overnight. The absorbance was read after 24 h. When higher MIC values are observed in the presence of sorbitol than in the absence of sorbitol this implicates the cell wall as one of the possible cell targets for the fungi [33–35].

### 2.8. Determination of the Effects of Extracts on Membrane Integrity: Nucleic Acid Leakage

The effect of the extract on *C. tropicalis*, *M. aurum,* and *M. smegmatis* was determined using the protocol of El-Nakeeb et al. [36]. Cells were grown overnight in a conical flask. Cells were centrifuged at 3500 rpm for 10 min and standardized to an optical density (OD) of 1.5 at 600 nm in 0.9% saline water. The cells were exposed to 1% sodium dodecyl sulfate, the standard drug (miconazole/rifampicin), MIC, 2 × MIC concentrations of ethyl acetate, and 70% ethanolic aqueous and incubated at 37°C for 10 min. An aliquot of 1 mL was taken from each tube after incubation and centrifuged in 2 mL microcentrifuge tubes at 10,000 rpm for 1 min (Centrifuge 5415C, Berlin, Germany). The supernatant was discarded, and the pellet was washed in 0.9% saline followed by repeated centrifugation. The pellet was resuspended in 0.9% saline, and 3 μL of PI was added to the cells and the tubes were kept in the dark for 10 min. PI is a dye used for bacterial viability staining. This dye can only cross disrupted or damaged bacterial membranes and is therefore considered an indicator of the integrity of the membrane. It stains nucleic acids inside dead cells or cells with a reversibly damaged membrane [37]. An *f*_max_ spectrofluorometer was used to measure fluorescence at an excitation wavelength 544 nm and emission 612 nm (Molecular Devices, Sunnyvale, USA).

### 2.9. Determination of the Effects of Potent Extracts on Membrane Integrity: Protein Leakage

The effect of the extracts on protein leakage in *C. tropicalis*, *M. aurum*, and *M. smegmatis* was determined using the protocol described in [36]. The cells were grown for 20 h in a 300-mL conical flask, incubated in a shaker at 100 rpm, centrifuged at 3500 rpm for 10 min, and standardized to an OD of 1.5 at 600 nm in 0.9% saline water. The cells were exposed to 0.1% sodium dodecyl sulfate, standard drug (miconazole/rifampicin), MIC, 2 × MIC concentrations of ethyl acetate, and 70% ethanolic aqueous and incubated at 37°C, 120 rpm for 120 min. Aliquots of 50 μL were taken from each tube, and 450 μL of 0.5 M NaOH was added followed by 1.5 mL of Bradford reagent. The Bradford assay relies on the binding of the dye Coomassie Blue G250 (Brilliant blue) to protein. The quantity of protein can be estimated by determining the amount of dye in the blue ionic form [38]. The samples were incubated for 10 min at room temperature, and the microplate reader was used to read the absorbances at 590 nm (Tecan Genios-Pro microplate reader, Grödig, Austria).

### 2.10. Statistical Analysis

The results were analyzed using a one-way analysis of variance test (ANOVA) with Dunnett's multiple comparison posttest. All datasets were compared to the control group, and any values with a *p*-value less than 0.05 were deemed statistically significant. GraphPad Prism 8 Software (Version 8.0.2, GraphPad Software Inc., San Diego, USA) was used for both graphical and statistical analyses.

## 3. Results

### 3.1. GC–MS Analysis

**Figure 1:**
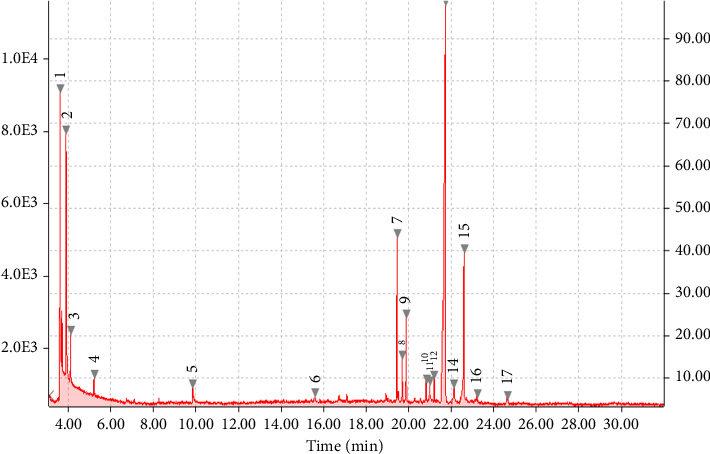
Total ion chromatogram (TIC) produced from 17 major peaks for compounds detected from the ethyl acetate leaf extract of *T. emetica*.

**Figure 2:**
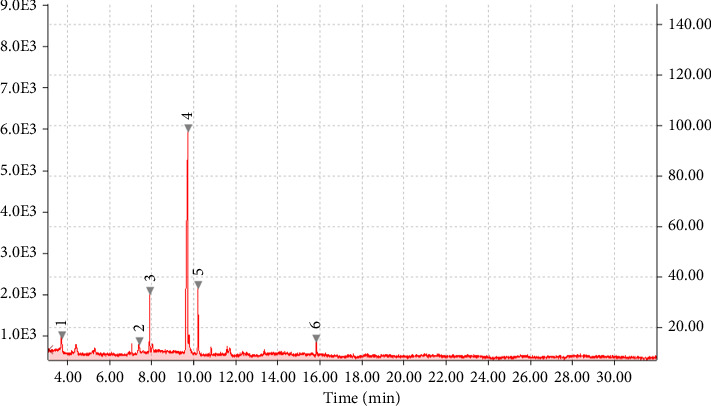
Total ion chromatogram (TIC) produced from 6 major peaks for compounds detected from the 70% ethanolic aqueous leaf extract of *T. emetica*.

Chemical profiling of *T. emetica* leaf extracts revealed the presence of a total of seven phytochemicals in both the ethyl acetate and 70% ethanol extracts. Figures 1 and 2 depict the GC-MS chromatograms of the *T. emetica* leaf extracts. However, the chromatograms also contain peaks corresponding to compounds that were identified as synthetic compounds. For the ethyl acetate extract, seven peaks were identified as phytochemicals, and out of those seven peaks, two were duplicates of compounds found in other extracts. This meant that five compounds were identified, namely gamabufatolin, 9-octadecen-1-ol(Z), octadecanoic acid, Rescinnamine, and cis-9-tetradecen-1-ol. In the 70% ethanol extract, two phytochemicals were identified: toluene and 2,4-di-tert-butylphenol. These compounds represent a diverse range of classes, including bufadienolides, fatty alcohols, fatty acids, alkaloids, aromatic hydrocarbons, and phenolic compounds. Based on retention time, the phytochemicals identified are shown in Tables 1 and 2.

### 3.2. Determination of MICs and MBCs/MFCs

**Figure 3:**
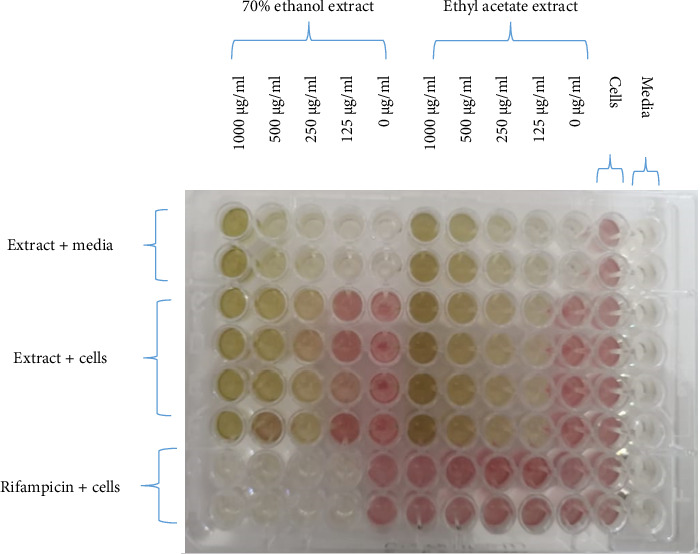
Viability assessment test of *T. emetica* ethyl acetate and 70% ethanolic aqueous leaf extracts against *M. aurum* using INT. Cells were grown in broth and exposed to increasing concentrations of extracts in a 96-well plate. Cells in media row were the negative control, while the cells in rifampicin row were the positive control. The wells before the color change represented the MIC of the extracts against the test microorganism.

**Figure 4:**
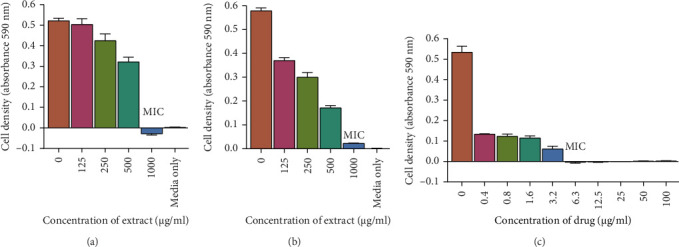
The effect of extracts on *C. albicans*. *C. albicans* was grown in broth and exposed to 70% ethanolic aqueous (a) and ethyl acetate (b) leaf extracts with increasing concentrations on a 96-well plate. Media was used as the negative control and miconazole (c) was used as the positive control. The cells were incubated in a shaking incubator at 37°C for 20 h. Absorbance was read using a microplate reader at 590 nm. A general increase in inhibition of cell growth is observed as the concentration of extracts increases. Each dataset represents the arithmetic mean *n* = 4.

**Figure 5:**
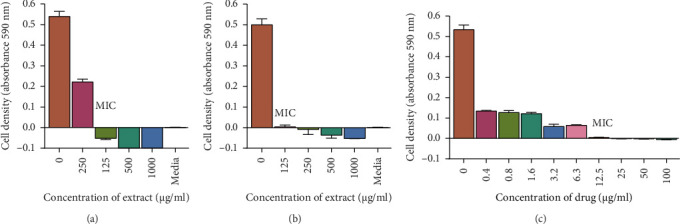
The antifungal activity of *T. emetica* extracts on *C. tropicalis*. *C. tropicalis* cells were grown in Sabouraud broth and exposed to 70% ethanolic aqueous (a) and ethyl acetate (b) leaf extracts with increasing concentrations on a microtiter plate. Sabouraud broth was used as the negative control and miconazole (c) was used as the positive control. The incubation of the cells was done in a shaking incubator at 37°C for 20 h. Absorbance was read using a microplate reader at 590 nm. An increase in inhibition of cell growth was observed with increase in concentration of extracts and the MFC of the 70% ethanolic aqueous and ethyl acetate leaf extracts was 1000 μg/mL. The values represent the means of 2 samples analyzed individually in quadruplicate.

**Figure 6:**
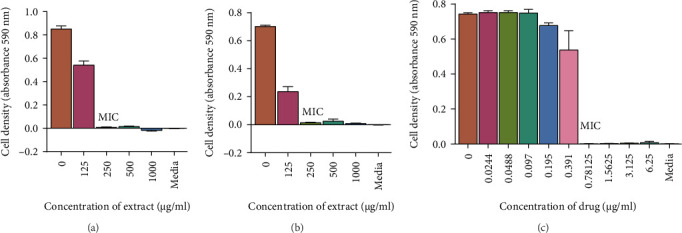
The antimycobacterial activity of extracts on *M. aurum.* The mycobacterial cells were grown in Middlebrook broth and treated with different concentrations of 70% ethanolic aqueous (a) and ethyl acetate (b) leaf extracts. Middlebrook media containing DMSO was used as the negative control and rifampicin (c) was used as the positive control. The treated cells were incubated at 37°C for 20 h. A microplate reader was used to read the absorbance at 590 nm. The MICs of the ethyl acetate and 70% ethanolic aqueous extracts were 250 μg/mL. All values represent mean *n* = 4.

**Figure 7:**
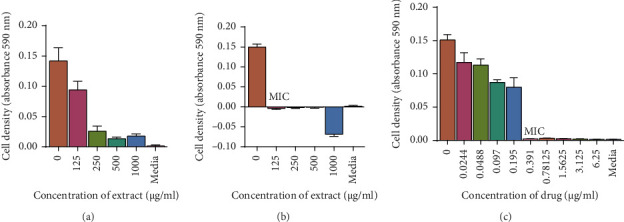
Growth inhibitory activity of extracts on *M. smegmatis.* The MIC of the ethyl acetate extract was 125 *μ*g/mL. Mycobacterial cells were treated with 1000, 500, 250, and 125 *μ*g/mL of 70% ethanolic aqueous (a) and ethyl acetate (b) leaf on a 96-well plate. Middlebrook broth was used as the negative control and rifampicin (c) was used as the positive control. The 96-well plate was placed in a 37°C shaking incubator for 20 h. Absorbance was read using a microplate reader at 590 nm. The *x*-axis represents the concentrations of extracts, and the *y*-axis represents cell density. Each dataset represents the arithmetic mean *n* = 4.

A visual representation of the microplate layout used in the microbroth assay for determining MICs is shown in Figure 3. The extracts exhibited antimicrobial activity with MICs ranging from 125 μg/mL to 1000 μg/mL as shown in Table 3. However, the water extract did not possess any antimycobacterial or antifungal activity against the tested microorganisms. The acetone extract did not possess any antifungal activity against *C. albicans* and *C. tropicalis.* The most potent extracts were the ethyl acetate and the 70% ethanolic aqueous extracts. The MICs of the two extracts were 125 μg/mL for *M. smegmatis*, 250 μg/mL for *M. aurum*, and 1000 μg/mL for *C. albicans*. A graph representing the activity of the two most potent extracts against *C. albicans* is also shown in Figure 4. The MICs for the ethyl acetate extract against *C. tropicalis* and 70% ethanolic aqueous extract were 250 μg/mL and 125 μg/mL, respectively, as shown in Figure 5. MBC and MFC were determined only for the most potent extracts. Values are for mean ± standard deviation (error bar) for *n* = 4. The MBC of the extracts against *C. albicans* was greater than 1000 μg/mL. The MICs of the extracts against the other 2 test organisms ranged from 250 to 500 μg/mL as shown in Figures 6 and 7.

### 3.3. Determination of the Effects of Ethyl Acetate and 70% Ethanolic Aqueous Extract on the Fungal Cell Wall: Sorbitol Assay

**Figure 8:**
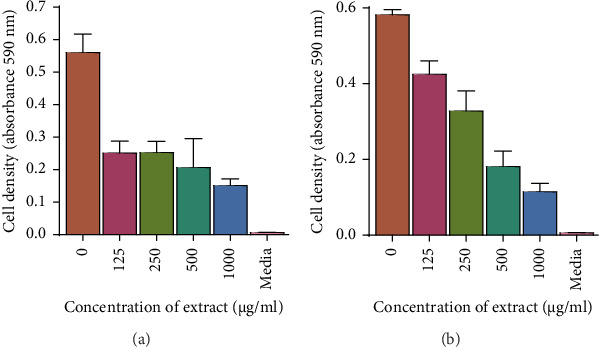
*C. tropicalis* was grown in broth and exposed to 1000, 500, 250, and 125 *μ*g/mL of the leaf extracts of ethyl acetate (a) and 70% ethanolic aqueous (b). Sterile broth was used as the negative control and miconazole was used as the positive control. The cells were incubated in a shaking incubator at 37°C for 20 h. Absorbance was read using a microplate reader at 590 nm. The MICs increased to greater than 1000 μg/mL when *C. tropicalis* was grown in the presence of extract and sorbitol. Each dataset represents the arithmetic mean *n* = 4.

The results showed that the antifungal properties of the ethyl acetate and 70% ethanolic aqueous extracts are related to the biosynthetic pathways of the cell wall because the MICs of the antifungal test changed in the presence of an osmotic protector compared to the absence of the osmotic protector. Initially in the absence of sorbitol, the MIC of the ethyl acetate and hydro ethanolic extracts was 250 and 125 μg/mL, respectively. As shown in Figure 8, the MIC values of the extracts against *C. tropicalis* increased in the presence of sorbitol to greater than 1000 μg/mL.

### 3.4. Determination of the Effects of Ethyl Acetate and 70% Ethanolic Aqueous Extract on Membrane Integrity: Nucleic Acid Leakage Assay

**Figure 9:**
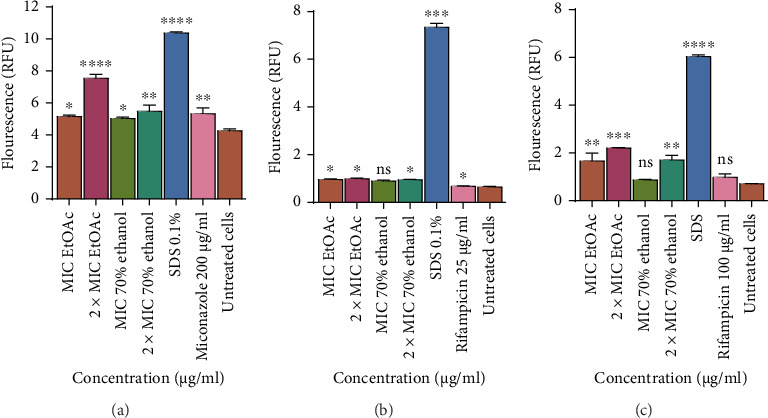
The effect of ethyl acetate and 70% ethanolic aqueous leaf extracts from *Trichilia emetica* on the cell membrane of (a) *C. tropicalis*, (b) *M. smegmatis*, and (c) *M. aurum* after exposure to the ethyl acetate and 70% ethanolic aqueous leaf extracts of *Trichilia emetica*. Cells that were not treated with extract were used as the control. The fluorescence values of the propidium iodide that is bound to nucleic acids are for mean ± standard deviation (error bar) for *n* = 2. The asterisks indicate a significant difference from the control with ⁣^∗∗∗^*p* < 0.001. Most of the extracts caused nucleic acid leakage in *C. tropicalis* and *M. smegmatis* but not for *M. aurum*. ⁣^∗^*p* < 0.05; ⁣^∗∗^*p* < 0.01⁣^∗∗∗∗^*p* < 0.0001 ns = not significant.

The effect of the 70% ethanolic aqueous and ethyl acetate (EtOAc) extract on nucleic acid leakage was determined by the fluorescence from the PI dye bound to nucleic acid inside damaged cells as shown in Figure 9. The concentrations used were MIC 125 μg/mL; 2 × MIC 250 μg/mL and MIC 250 μg/mL; 2 × MIC 500 μg/mL, respectively. Nucleic acid was observed to increase as the concentration of the extract increased. The extracts caused nucleic acid leakage in *C. tropicalis,* which was observed because the fluorescence in the MIC was 5.03 f/units and 2 × MIC was 7.55 f/unit as compared to the SDS which had 10.38 f/unit. There was lower nucleic acid leakage in *M. smegmatis* as the fluorescence was 0.87 f/units for the MIC and 2.21 f/units for the 2 × MIC as compared to SDS, which had 6.037 f/unit. However, there was no nucleic acid leakage caused by the extracts in *M. aurum.* Values are for mean ± standard deviation (error bar) for *n* = 2.

### 3.5. Determination of the Effects of Ethyl Acetate and 70% Ethanolic Aqueous Extract on Membrane Integrity: Protein Leakage Assay

**Figure 10:**
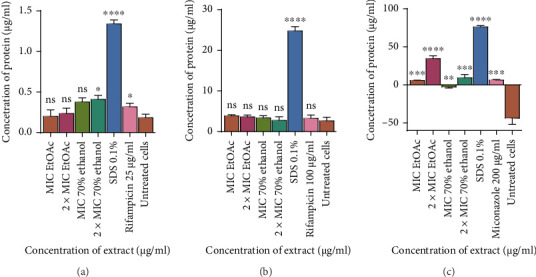
Leakage of proteins from (a) *M. aurum*, (b) *M. smegmatis*, and (c) *C. tropicalis* after exposure to *Trichilia emetica* ethyl acetate and 70% ethanolic aqueous leaf extracts. As a control, cells that were not exposed to the extract were used. The values represent the mean ± standard deviation of samples *n* = 2. One-way ANOVA was used to analyze the results and the asterisks indicate statistical differences from the control (⁣^∗∗∗^*p* < 0.001). Leakage of proteins from *M. aurum* and *C. tropicalis* was dependent on the increase in the concentration of the extracts. ⁣^∗^*p* < 0.05; ⁣^∗∗^*p* < 0.01⁣^∗∗∗∗^*p* < 0.0001 ns = not significant.

The effect of the 70% EtOAc extract on protein leakage was determined by quantifying protein from the cells after exposing them to MIC 125 *μ*g/mL; 2 × MIC 250 *μ*g/mL and MIC 250 *μ*g/mL; 2 × MIC 500 *μ*g/mL, respectively. As shown in Figure 10, the extracts did not cause significant protein leakage in *M. smegmatis* as compared to SDS. The extracts did not cause protein leakage in *M. aurum* except for 2 × MIC which caused little leakage. However, there was protein leakage caused by ethyl acetate MIC; 2 × MIC and 70% ethanolic aqueous MIC; 2 × MIC of 5.80, 36.0, 2.16, and 7.51 *μ*g/mL, respectively, against C. *tropicalis*. The amount of nucleic acids which were leaked was observed to be dependent on the concentration of the extract. Values are for mean ± standard deviation (error bar) for *n* = 2.

## 4. Discussion

Candida species are some of the yeasts that cause severe infections, hence, causing significant morbidity and mortality in affected patients. These effects of Candida have encouraged the exploration of new therapeutic approaches to treat candidiasis [49]. Several Candida species are referred to as opportunistic pathogens because they are found in the normal microbiota and only cause harm in immunosuppressed individuals [50]. The treatment of mycobacterial strains that are drug resistant has become a major global problem [51]. Mycobacterial species are ubiquitous; thus, they are easily in contact with humans. In many cases, pathogenic mycobacteria can breach the innate system and cause soft tissue and respiratory infections [52]. Plants are an important source of drugs as they have a wide diversity of molecules that have medicinal properties and, therefore, can greatly contribute to the search for novel bioactive products or lead compounds for the development of therapeutic agents [53]. In this study, *T. emetica* was selected as it has been used in Africa to treat different diseases, including respiratory and skin infections [18]. The antimycobacterial and antifungal potential of the extracts was evaluated against *M. smegmatis*, *M. aurum*, *C. tropicalis,* and *C. albicans.*

Microextraction was used to prepare the extracts. Successful evaluation of bioactivity of extracts is based on the solvent and extraction method used. Polar, medium polar, and nonpolar solvents were used to have a wide range of phytochemicals in the extracts ranging from polar to nonpolar extracts [54]. Ethyl acetate and 70% ethanolic aqueous extract were the most potent against *M. smegmatis*, *M. aurum*, *C. albicans,* and *C. tropicalis*. The high activity shown by the 70% ethanolic aqueous extract against all the test organisms is because ethanolic solvents are more efficient in entering cell walls causing the release of constituents such as polyphenols [55–57]. The 70% ethanolic aqueous extract yields higher concentrations of bioactive flavonoids as 70% ethanolic aqueous has higher polarity than pure ethanol [58]. In addition, they penetrate the cell membranes to extract intracellular components from the leaf sample. Ethyl acetate has medium polarity, meaning both polar and nonpolar phytochemicals are extracted, which could have influenced bioactivity. The exhibited bioactivity makes *T. emetica* a possible source of compounds for drug development.

Five naturally occurring compounds were identified using the GC-MS, namely gamabufatolin, 9-octadecen-1-ol(Z), octadecanoic acid, Rescinnamine, and cis-9-tetradecen-1-ol. In the 70% ethanol extract, two phytochemicals were identified: toluene and 2,4-di-tert-butylphenol. In fungi such as *Candida* species, the role of the fungal membrane is to maintain the integrity and order of the cells. This is why, most antifungal treatments mainly target the fungal membrane [59]. Fatty acids directly interact with the cell membranes of fungi, and this has been determined as the mode of action. These fatty acids are able to insert themselves into the fungal membrane, thereby, physically destabilizing the fluidity of the membrane. The elevation in fluidity of the membrane causes conformational changes of the membrane proteins, leakage of intracellular components cytoplasmic disorder, and eventually cell death [60]. Antifungal free fatty acids can be either saturated or unsaturated. The effectiveness of these fatty acids against fungi tends to increase with longer carbon chain lengths [61]. The hydrophobic properties of saturated fatty acids are particularly pivotal for their bioactivity [62]. Lauric, palmitic, stearic, and myristic acids have demonstrated significant potential as antibacterial and antifungal agents [63]. Octadecanoic acid may have contributed to the antifungal activity observed in plant extracts, as evidenced by its detection in the GC–MS analysis of the extract. This fatty acid also has antiviral properties and low cytotoxicity levels against mammalian cells. It has been reported to have inflammatory, hypocholesterolemic, hepatoprotective, nematicidal, insectifuge, antihistamine, antieczemic, and antiacne activity [64, 65].

Yuan et al. [66], demonstrated that gamabufotalin is a bufadienolide compound. Bufadienolides are a major class of compounds secreted from the skin of amphibian. They are characterized by the presence of *α*-pyrone ring and fall under the class of cardiac steroids. They are prescribed as antitumor agents for people suffering from cardiovascular diseases [67]. Bufadienolides are effective constituents of cinobufacini and have been used to treat cancers such as hepatoma, gallbladder, carcinoma, and lung cancers. The compounds have been reported to have cytocidal effects on cancers and minimum cytotoxicity to mammalian cells. They also have antitumor immunity [66]. In a study by Susanti et al. [68], cis-9-tetradecen-1ol has been identified from an essential oil isolated from the flowers of Etlingerea elatoir. Cis-9-tetradecen-1-ol has been identified as pheromone in *Spodoptera eridania* and *Argyrotaenia ljungiana* [69]. Z-9 octadecen-1-ol was identified as a natural aphrodisin in hamsters [70]. This sex pheromone has also been identified in *Simmondsia chinensis* [42]. Triterpenes and sterols have the ability to increase cell permeability and loss of cellular components from microbial cells [71]. Several limonoids have been isolated from the bark and seeds of *T. emetica*, and most of the isolated compounds have the common name trichilin [72, 73]. Limonoids are modified triterpenes with a structure which either contains or is derived from a precursor with a 4,4,8-trimethyl-17-furanyl steroid skeleton [74]. Pagna et al. [75] reported that the presence of limonoids in *T. emetica* can be considered responsible for the activity against clinical bacterial strains. The arrangement of ring structures in limonoids provides the characteristics that generate the interesting aspect of this plant. These characteristics include medicinal properties such as antibacterial, antifungal, and antiviral [76].

The differences in the current study and other studies could be the difference between extraction processes. In other studies, the extracts of the leaf and seed of *T. emetica* had antifungal activity. Methanol fruit extract (500 *μ*g/mL) of *T. emetica* slowed the growth of *C. albicans, Trichophyton violaceum, Cryptococcus neoformans, Aspergillus flavus*, and *Trichophyton mentagrophytes* [77]. Shai et al. [78] investigated the antifungal activity of acetone, *n-*hexane, and DCM leaf extracts against *C. albicans, C. neoformans, Aspergillus fumigatus, Sporothrix schenckii*, and *Macrococcus canis* using the micro dilution assay. In that study, it was shown that the hexane extract exhibited antifungal activity with an MIC (0.15 mg/mL) against *M. canis.* The DCM extract exhibited little activity against test pathogens compared to the other extracts [78]. Phytochemicals such as alkaloids and saponins present in the plant may be responsible for the antimycobacterial and antifungal activity [79, 80]. The waxy cell envelope is known to be innate to antimicrobial agents; however, mycobacterial cells were susceptible to the extracts [81].

Further evaluation was carried out to determine the effect of extracts on the cell wall and cell membrane of the test microorganisms. The mode of action was tested to determine whether the antifungal activity of the extracts involved a direct interaction with the structure of the *C. tropicalis* cell wall [33]. To investigate the action of the extracts on the cell wall of *C. tropicalis*, the sorbitol assay was performed. Sorbitol is a common osmotic protector that is used to stabilize fungal protoplasts. If sorbitol protects cells, they can grow in the presence of inhibitors of fungal cell wall synthesis, where growth would be inhibited in the absence of sorbitol. This is observed by an increase in the MIC value as compared to in the absence of sorbitol [34, 82]. In this study, the MIC values increased in the presence of sorbitol to more than1000 *μ*g/mL. This suggests that the extracts do inhibit the fungal cell wall synthesis and probably by affecting other cell targets.

The cell membrane is a structure that acts as a barrier between the cytoplasm and the extracellular medium [83]. Most antimicrobial agents usually act on membranes of microorganisms by causing disruption [84]. The test microorganisms were exposed to two extracts at MIC and 2 × MIC, and the leakage of nucleic acid was determined by adding PI to the cells and taking fluorescence readings. The ability of PI to bind to nucleic acid and fluoresce is the principle behind determining the leakage of nucleic acids. This dye is not able to enter live cells; hence, it can only enter damaged cells via damaged cell membranes [85]. PI is a universal indicator of cell death because the loss of integrity of the plasma membrane is a common event in all forms of cell death [86]. The ethyl acetate extract caused significant leakage of nucleic acid compared to 70% ethanolic aqueous in *C. tropicalis*. This could be due to the difference in polarities between the two extracts. The ethyl acetate extract caused damage to the cell membrane of *M. smegmatis* and *C. tropicalis.* However, there was no damage to the cell membrane of *M. aurum*. Sodium dodecyl sulfate, which is a surfactant, was used as the control. Surfactants achieve cell lysing by interfering with cell membranes. Surfactants have similarities to membrane phospholipid molecules; therefore, they can adsorb at the cell membrane and disrupt it [87, 88]. Standard drugs used did not cause any significant leakage, as they are already known to target parts of the microorganisms that are not the cell wall or cell membrane. In a study by Mautsa and Mukanganyama [89], the ethyl acetate leaf extract of *V. adoensis* caused membrane disruption and damage to *M. smegmatis.* This could possibly mean that ethyl acetate extracts phytochemicals that can disrupt the membrane of *M. smegmatis*.

The inability of the extract to cause leakage of proteins from *M. smegmatis* suggests that the extract is not capable of disrupting the integrity of the mycobacterial cell membrane. This means that the extract has another part of the cell that it targets, which is not the cell membrane. Since the two extracts have phytochemicals of different polarities, the 70% ethanolic aqueous extract managed to cause the leakage of proteins at 2 × MIC, whereas the ethyl acetate did not cause leakage. However, the two extracts managed to cause the leakage of proteins in *C. tropicalis* meaning that the extracts can disrupt membrane integrity.

## 5. Conclusions

The leaf extracts of *T. emetica* have antifungal activity against *C. albicans* and *C. tropicalis*. It was also observed that they have antimycobacterial activity against *M. smegmatis* and *M. aurum.* The GC–MS profile showed the presence of fatty acids, fatty alcohols, bufadienolides, alkaloids, aromatic hydrocarbons, and phenolic compounds. The fatty acids and fatty alcohols may be responsible for the antimycobacterial and antifungal activity. These findings support the traditional use of *T. emetica* leaves to treat respiratory and skin infections. This plant can, therefore, be used to provide new compounds for the development of antimicrobial agents. Further investigation should be done to determine the cytotoxicity of the extracts on cancer and normal mammalian cells.

## Figures and Tables

**Table 1:** The main proposed compounds identified using the NIST17 library from *T. emetica* using ethyl acetate leaf extract.

Name of compound and molecular formula	Molecular structure	RT (min)	Function
Gamabufotalin C_24_H_34_O_5_	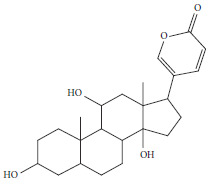	5.21	Anticancer [39]

*cis*-9-Tetradecen-1-ol C_14_H_28_O	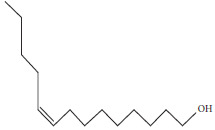	9.698	Sex pheromones [40]

9-Octadecen-1-ol, (Z)- C_18_H_36_O	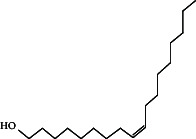	19.887	Sex pheromones [41]
			Insecticidal [42]

Octadecanoic acid C_18_H_36_O_2_	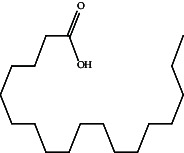	20.997	Antibacterial [43]
			Anticancer [44]
			Antifungal [45]

Rescinnamine C_35_H_42_N_2_O_9_	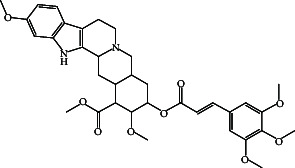	22.13	Antihypertensive [46]

**Table 2:** The main proposed compounds identified using the NIST17 library from *T. emetica* using 70% ethanolic aqueous leaf extract.

Name	Molecular structure	RT (min)	Function
Toluene C_7_H_8_	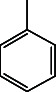	3.716	Nematicidal [47]

2,4-Di-tert-butylphenol C_14_H_22_O	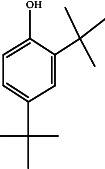	15.818	Antioxidant [48]
			Anticancer [48]

**Table 3:** Minimum inhibitory concentrations and minimum fungicidal/bactericidal concentrations of extracts against *M. smegmatis, M. aurum, C. albicans*, and *C. tropicalis*.

Fraction of plant extract	*M. smegmatis*		*M. aurum*		*C. albicans*		*C. tropicalis*	
	MIC	MBC	MIC	MBC	MIC	MBC	MIC	MBC
	(μg/mL)		(μg/mL)		(μg/mL)		(μg/mL)	
Hexane	500		500		—		500	
Dichloromethane	500		1000		—		500	
Ethyl acetate	125	> 1000	250	250	1000	> 1000	250	250
Acetone			1000		—		—	
Ethanol	1000		500		—		1000	
Methanol	500		1000		—		1000	500
Water	—		—		—		—	
70% ethanolic aqueous	125	250	250	500	1000	> 1000	250	250
DCM:methanol	1000		1000		—		1000	

## Data Availability

The datasets generated during the current study are available from the corresponding author upon reasonable request.
